# When Problems Only Get Bigger: The Impact of Adverse Childhood Experience on Adult Health

**DOI:** 10.3389/fpsyg.2021.693420

**Published:** 2021-07-14

**Authors:** Márcia Novais, Teresa Henriques, Maria João Vidal-Alves, Teresa Magalhães

**Affiliations:** ^1^School of Medicine, Universidade do Porto (University of Porto), Porto, Portugal; ^2^Department of Community Medicine, Information and Health Decisions, School of Medicine, University of Porto, Porto, Portugal; ^3^Center for Research in Health Technologies and Services, School of Medicine, University of Porto, Porto, Portugal; ^4^Department of Public and Forensic Health Sciences and Medical Education, School of Medicine, University of Porto, Porto, Portugal; ^5^University Institute of Health Sciences - CESPU, Gandra, Portugal

**Keywords:** child abuse, trauma, adverse childhood experience, health, risk behavior

## Abstract

**Introduction:** Previous studies have shown that adverse childhood experiences negatively impact child development, with consequences throughout the lifespan. Some of these consequences include the exacerbation or onset of several pathologies and risk behaviors.

**Materials and Methods:** A convenience sample of 398 individuals aged 20 years or older from the Porto metropolitan area, with quotas, was collected. The evaluation was conducted using an anonymous questionnaire that included sociodemographic questions about exposure to adverse childhood experiences, a list of current health conditions, questions about risk behaviors, the AUDIT-C test, the Fagerström test and the Childhood Trauma Questionnaire–brief form. Variables were quantified to measure adverse childhood experiences, pathologies, and risk behaviors in adult individuals for comparison purposes.

**Results:** Individuals with different forms of adverse childhood experiences present higher rates of smoking dependence, self-harm behaviors, victimization of/aggression toward intimate partners, early onset of sexual life, sexually transmitted infections, multiple sexual partners, abortions, anxiety, depression, diabetes, arthritis, high cholesterol, hypertension, and stroke. Different associations are analyzed and presented.

**Discussion and Conclusions:** The results show that individuals with adverse childhood experiences have higher total scores for more risk behaviors and health conditions than individuals without traumatic backgrounds. These results are relevant for health purposes and indicate the need for further research to promote preventive and protective measures.

## Introduction

Child maltreatment (MT) or child abuse and neglect is a severe public health problem with a long-term impact on the victim's life and health (Felitti et al., 2019). Child maltreatment, along with other types of trauma, is currently considered an adverse childhood experience (ACE). Felitti et al. (2019) took the first steps to designate MT categories as an ACE [“Centers for Disease Control and Prevention. Preventing adverse childhood experiences (ACEs): leveraging the best available evidence,” 2019; Felitti et al., 2019]. Seven categories of ACEs were initially studied: three were related to child MT (physical, sexual, and psychological abuse), and four family dysfuncion (substance abuse, mental illness, MT of the mother, and criminal behavior) [Andersen and Blosnich, 2013; “Centers for Disease Control and Prevention. Preventing adverse childhood experiences (ACEs): leveraging the best available evidence,” 2019; Felitti et al., 2019]. In addition to the MT itself, ACEs' cumulative effect has also been highlighted. A dose-effect relationship has been identified between the number of ACEs and the risk of a higher incidence of negative outcomes (Chapman et al., 2004; Felitti et al., 2019).

The consequences of MT can be immediate or can arise in the medium or long term (Flaherty et al., 2013; Chiang et al., 2015; Kalmakis and Chandler, 2015). The most frequent and immediate effects are traumatic injuries (e.g., bruises, abrasions, hematomas, fractures, traumatic brain injury) and psychosomatic disorders associated with stressful experiences (e.g., sleep or eating disorders, anxiety disorder). In the medium and long term (in youth and adulthood), health risk behaviors and physical and mental health disorders may arise (Larkin et al., 2012; King, 2020).

Regarding health risk behaviors, there is a trend on increased rates of substance abuse (Hillis et al., 2001; Dube et al., 2003; Andersen and Blosnich, 2013; Felitti et al., 2019) (e.g., abuse of anxiolytics, analgesics, tobacco, drugs, and alcohol), physical inactivity (Hillis et al., 2001; Felitti et al., 2019), risky sexual behaviors (Dube et al., 2003; Andersen and Blosnich, 2013; Felitti et al., 2019) (e.g., juvenile sexuality, multiple partners, unprotected intercourse), self-harming behaviors (Dube et al., 2003; Andersen and Blosnich, 2013) (e.g., self-aggression and attempted suicide or suicide), and deviant behaviors related to repeated violence (e.g., intimate partner aggression) (Hillis et al., 2001; Dube et al., 2003; Andersen and Blosnich, 2013).

These consequences are associated with the impact of traumatic stress on various systems linked to body homeostasis (namely, the neurological, immune, and endocrine systems), including the hypothalamic-pituitary-adrenal (HPA) axis. The hyperactivation of this axis favors the occurrence, in the long term, of dysfunctions at different levels: metabolic (Vargas et al., 2016; Felitti et al., 2019) (e.g., obesity, dyslipidaemia, diabetes); cardiocerebrovascular (Dong et al., 2004; Stein et al., 2010; Andersen and Blosnich, 2013) [e.g., atherosclerosis, high blood pressure (HBP), acute myocardial infarction, stroke]; respiratory (Anda et al., 2008; Bellis et al., 2015; Hughes et al., 2017; Felitti et al., 2019) (e.g., chronic obstructive pulmonary disease); inflammatory (Danese et al., 2007; Dube et al., 2009) (e.g., rheumatoid arthritis, lupus, asthma); oncological (Holman et al., 2016; Felitti et al., 2019) and sexual/reproductive [sexually transmitted infections (STI), unwanted pregnancy and abortions] (Hillis et al., 2001; Dube et al., 2003; Andersen and Blosnich, 2013; Felitti et al., 2019). Increased cortisol levels, decreased activity of cortisol receptors and increased corticotrophin-releasing factor (Dube et al., 2003; Anda et al., 2006) can trigger genetically programmed diseases that would be latent in the absence of traumatic stress (Dube et al., 2003; Anda et al., 2006). On the other hand, a change in the HPA axis may also contribute to a pro-inflammatory state and a consequent increase in morbidity such as depressive disorders (Heim et al., 2008), rheumatoid arthritis (Colebatch and Edwards, 2011), and metabolic diseases, such as hyperinsulinemia (Maniam et al., 2014).

It is, however, important to note that these outcomes have individual variability. They are influenced by age, resilience, personality characteristics, and developmental stage that the victim was at when MT occurred, type, frequency, duration, and severity of MT, and by the victim-abuser relationship/closeness (Leitch, 2017). Their occurrence also depends on the early detection of MT and the quality of professional intervention received. So, not all adult individuals who have suffered ACEs–specifically MT–will have negative health outcomes, yet, a great proportion is expected to be at increased risk for such (Monnat and Chandler, 2015). Thus, it is currently understood that traumatic experiences in childhood may lead to many disorders and pathologies that manifest in adulthood, many of which may have gone unnoticed in health contexts during childhood (Kalmakis and Chandler, 2015).

This study's general objective is to analyze the relationship of ACEs, namely, those related to MT, with the occurrence of adult health problems in a population in the Porto region, in Portugal.

## Materials and Methods

The present study was based on self-completed questionnaires administered to a convenience sample of participants collected according to quotas, stratified by gender and age and drawn from the Porto region's population (1,391,726 inhabitants). The total sample consisted of 398 healthcare users who met the following selection criteria: (a) residents of the Porto area and (b) older than 19 years old, taking into account the age group for which the childhood trauma questionnaire that we used [CTQ-SF] was validated. A member of the research team distributed paper versions of the questionnaire in five private healthcare services (medical centers and clinics) in the Porto area. In each center/clinic, an internal collaborator recruited participants and managed both delivery and reception of questionnaires, using a deposit box in order to guarantee the anonymity of participants. Responses were collected between July 2019 and January 2020. The questionnaire included information about the participants, reflecting the literature on the topic (Nabais, 2014; Alves et al., 2015): (a) sociodemographic data, (b) risk behaviors (alcohol consumption, tobacco use, drug abuse, onset of sexual activity before 16 years of age, three or more sexual partners in the last year, abortion before age 18 years, attempted suicide, self-harm behaviors, being aggressive to an intimate partner, being victimized by an intimate partner), and (c) current health conditions (hypercholesterolemia, HBP, anxiety disorder, diabetes, depression, asthma, rheumatoid arthritis, thyroid pathology, stroke sequelae, oncological disease, liver disease, renal pathology, acute myocardial infarction sequelae, chronic obstructive pulmonary disease, or bulimia).

Sociodemographic data included age, sex and level of education of the respondents. This last parameter was classified as (a) elementary school completion (b) high school attendance (c) high school completion and (d) higher education attendance or completion. The referred setting–private health services–is predominantly used by medium-high-income individuals (Nurses, 2011).

The AUDIT-C test, adapted for the Portuguese population (Cunha, 2002) from the work of Bush et al. (1998), was used for alcohol consumption screening. This tool is used to reliably identify people with hazardous drinking habits (i.e., binge drinking) or with active alcohol-related disorders (i.e., dependence).

The Fagerström test evaluated nicotine dependence using the validated version for the Portuguese population (Ferreira et al., 2009) from the scale by Heatherton et al. (1991), which showed satisfactory total internal consistency (α = 0.66) (Heatherton et al., 1991; Ferreira et al., 2009).

The CTQ-SF, in the validated version for the Portuguese population (Pennebaker and Susman, 1988; Dias et al., 2013) was employed to assess the individual's ACEs. This version is based on the short-form scale tested by Thombs et al. (2009) and its original version (Bernstein et al., 1998). The CTQ-SF includes 28 items rated on a 5-point Likert scale and has 5 main factors: *emotional* (α = 0.71), *physical* (α = 0.77) and *sexual* (α = 0.71) *abuse* and *emotional* (α = 0.79) and *physical* (α = 0.47) *neglect*. The total scale shows high internal consistency (α = 0.84). The reliability observed for the *physical neglect* subscale does not affect the total consistency of the scale, and its removal was not considered beneficial according to the confirmatory factor analysis performed by the authors in a non-clinical population (test-retest reliability) (Dias et al., 2013). Responses ranged from 1–never to 5–always and each of the five factors of the scale encompassed 4 to 7 items. Each of the 5 factors of the scale ranges from 5 to 25 points.

Statistical analysis was performed using the Statistical Package for Social Sciences (SPSS) version 26. We performed a descriptive analysis of the variables, and the categorical variables are described as absolute frequencies and their respective percentages. Chi-square tests were used to assess relationships between categorical variables. We first verified the distributions' normality for continuous variables, by observing the histogram; when normality was not observed, the continuous variables are described as medians and their respective interquartile ranges. Associations between the different ACE factors (*emotional abuse, physical abuse, sexual abuse, emotional neglect*, and *physical neglect*) were evaluated using Spearman's correlation. We used the Mann-Whitney and Kruskal-Wallis tests to compare the various ACE factors and different groups of risk behaviors and pathologies. The mean imputation technique was applied to the missing data in the CTQ-SF questions. A statistical significance level of 0.05 was considered.

The study was approved by the Ethics Committee for Health of the *Centro Hospitalar Universitário de São João* (University Hospital Center of São João) and the Medical School of the University of Porto.

## Results

### Respondent Characteristics

Of the respondents, 53.5% were female (*n* = 213). The mean age was 50.6 years (median = 50; min = 20; max = 97). Regarding education level, 14.8% (*n* = 59) completed elementary school, 15.8% (n = 63) attended high school without completing, 33.4% (*n* = 133) completed high school, and 35.7% (*n* = 142) had a higher education degree or were currently attending college.

At the time of the study, the respondents reported the following:

a) Regarding health risk behaviors: excessive alcohol consumption (*n* = 68; 24.0%); moderate and high consumption of tobacco (*n* = 86; 21.6%); drug abuse (*n* = 14; 3.5%); onset of sexual activity before 16 years of age (*n* = 72; 18.8% of 383 sexually active individuals); STI (*n* = 15; 3.9%); three or more partners in the last year (*n* = 14; 3.7%); abortion before age 18 years (*n* = 18; 4.7%); attempted suicide (*n* = 24; 6%); self-harm behaviors (*n* = 20; 5%); being aggressive to an intimate partner (*n* = 25; 6.3%); being victimized by an intimate partner (*n* = 15; 3.8%).
b) Regarding diseases: at the time of the study, 61.8% of the participants had at least one of the following diseases: hypercholesterolemia (*n* = 109; 27.4%), HBP (*n* = 93; 23.4%), anxiety disorder (*n* = 73; 18.3%), diabetes (*n* = 61; 15.3%), depression (*n* = 59; 14.6%), asthma (*n* = 36; 9.0%), rheumatoid arthritis (*n* = 23; 5.8%), thyroid pathology (*n* = 21; 5.3%), stroke sequelae (*n* = 20; 5.0%), oncological disease (*n* = 17; 4.3%), liver disease (*n* = 7; 1.8%), renal pathology (*n* = 7; 1.8%), acute myocardial infarction sequelae (*n* = 7; 1.8%), chronic obstructive pulmonary disease (*n* = 3; 0.8%) or bulimia (*n* = 1; 0.3%).

### ACE Factors and Their Correlations

Table 1 presents the median and interquartile ranges for the individual ACE factors and the general score (CTQ-SF) by gender and their correlations with the respondents' age. Bearing in mind the reliability previously found for the *physical neglect* subscale, the results obtained with this subscale in the present study were interpreted with due reserve. The *emotional neglect* factor had the highest values, meaning that a high percentage of respondents scored in this subscale, while *sexual abuse* and *physical abuse* had lower values and showed less variation among responses. Considering the sex of participants, *emotional abuse* and *sexual abuse* had higher scores among female respondents, although differences were moderate (*p* = 0.04). A significant negative association was found between age and emotional abuse in childhood, which means younger individuals report higher emotional abuse values. Also, a significant positive association was found between age and physical neglect, which means higher physical neglect values were more frequently reported by older respondents.

**Table 1:** ACE factors–median and interquartile range; median and interquartile range by gender; correlation with age.

	**Total (*n* = 398) Med^c^ (Q1^d^, Q3^e^)**	**Male (*n* = 185)Med^c^ (Q1^d^, Q3^e^)**	**Female (*n* = 213) Med^c^ (Q1^d^, Q3^e^)**	**Spearman's coefficient for age**
Emotional abuse	6 (5, 9)	**6 (5, 8)**	**7 (5, 9)**	**−0.143^a^**
Emotional neglect	8 (6, 11)	8 (6, 11)	8 (6, 11)	0.01
Sexual abuse	5 (5, 5)	**5 (5, 5)**	**5 (5, 5)**	−0.018
Physical abuse	5 (5, 6)	5 (5, 6)	5 (5, 6)	0.079
Physical neglect	6 (5, 9)	6 (5, 8)	6 (5, 9)	**0.300^b^**
CTQ-SF	32 (28, 38)	32 (28, 37)	32 (29, 38)	0.064

a *p < 0.005;*

b *p < 0.001;*

c *Med–median;*

d *Q1–first quartile;*

e *Q3–third quartile*.

*Values in bold indicate statistically significant results (p < 0.05)*.

There were three major associations among the different ACE factors. The strongest was between *emotional abuse* and *physical abuse* (*r* = 0.452), followed by the associations between *emotional abuse* and *emotional neglect* (*r* = 0.441) and between *emotional neglect* and *physical neglect* (*r* = 0.422) (Table 2). Since the Spearman correlations found between all of the CTQ-SF subscales are statistically significant (*p* < 0.01), this explains the shared variance. The *sexual abuse* subscale has the lowest correlation with the other subscales, as in the validation study. It was also found that the sum of the subscales' scores may be used as a general indicator of child maltreatment.

**Table 2:** Correlations between ACE factors.

	**Emotional neglect**	**Sexual abuse**	**Physical abuse**	**Physical neglect**	**CTQ-SF**
Emotional abuse	0.441^a^	0.372^a^	0.452^a^	0.235^a^	0.690^a^
Emotional neglect		0.204^a^	0.382^a^	0.422^a^	0.831^a^
Sexual abuse			0.296^a^	0.183^a^	0.367^a^
Physical abuse				0.382^a^	0.580^a^
Physical neglect					0.660^a^
CTQ-SF					

a *p < 0.001*.

### Health Risk Behaviors and Correlations With ACE Factors

No differences in alcohol and drug consumption were found among the various factors. Individuals with partial scores (higher scores for *emotional abuse, emotional neglect*, and *physical neglect*) and higher total scores had a higher dependence on tobacco (Table 3).

**Table 3:** Median, interquartile range, and *p*-value for the relationship between the different ACE factors and tobacco consumption.

			**Emotional abuse**	**Emotional neglect**	**Sexual abuse**	**Physical abuse**	**Physical neglect**	**CTQ-SF**
Tobacco consumption	NO (*n* = 250; 62.8%)	Med^a^ (Q1^b^,Q3^c^)	6 (5, 8)	9 (6, 11)	5 (5, 5)	5 (5, 6)	6 (5, 9)	33 (28, 38)
	YES (*n* = 86; 21.6%)	Med^a^ (Q1^b^,Q3^c^)	6 (5, 9)	8 (7, 11)	5 (5, 5)	5 (5, 6)	6 (5, 8)	31 (29, 37)
	No longer consumes (*n* = 62; 15.6%)	Med^a^ (Q1^b^,Q3^c^)	6 (5, 8)	7 (5, 10)	5 (5, 5)	5 (5, 7)	6 (5, 9)	32 (28, 38)
	KRUSKAL-WALLIS	*P*-value	0.459	0.129	0.193	**0.029**	0.404	0.729
Tobacco dependence	LOW (*n* = 62; 72.1%)	Med^a^ (Q1^b^,Q3^c^)	6 (5, 9)	7 (6, 10)	5 (5, 5)	5 (5, 6)	5 (5, 7)	30 (28, 34)
	MODERATE (*n* = 19; 22.1%)	Med^a^ (Q1^b^,Q3^c^)	7 (6, 9)	9 (7, 11)	5 (5, 5)	5 (5, 6)	6 (5, 9)	35 (30, 39)
	HIGH (*n* = 62; 5.8%)	Med^a^ (Q1^b^,Q3^c^)	14 (14, 19)	19 (15, 21)	5 (5, 9)	6 (5, 11)	16 (14, 17)	59 (56, 75)
	KRUSKAL-WALLIS	*P*-value	**0.013**	**0.008**	0.4	0.115	** < 0.001**	**0.002**

a *Med–median;*

b *Q1–first quartile;*

c *Q3–third quartile*.

*Values in bold indicate statistically significant results (p < 0.05)*.

Regarding high risk of sexual behaviors (Table 4): (a) individuals with early onset of sexual intercourse present higher scores on the *emotional abuse* factor and a significantly higher ACE total score; (b) individuals reporting prior STI reveal higher physical abuse scores; (c) individuals who reported not having a sexual partner in the last year or having had three or more, presented a higher score on the *physical neglect* factor; (d) individuals who reported having had at least one abortion before completing 18 years of age had higher scores on the *physical abuse* and *physical neglect* factors.

**Table 4:** Median and correlation between the different ACE factors and sexual risk behaviors.

			**Emotional abuse**	**Emotional neglect**	**Sexual abuse**	**Physical abuse**	**Physical neglect**	**CTQ-SF**
Age at onset of sexual intercourse, years	<16 (*n* = 72,18.8%)	Med^a^ (Q1^b^,Q3^c^)	7 (5, 10)	9 (7, 13)	5 (5, 5)	5 (5, 7)	7 (5, 9)	34 (30, 42)
	16–18 (*n* = 133, 34.7%)	Med^a^ (Q1^b^,Q3^c^)	6 (5, 8)	9 (6, 11)	5 (5, 5)	5 (5, 6)	6 (5, 8)	33 (28, 38)
	>18 (*n* = 171, 44.6%)	Med^a^ (Q1^b^,Q3^c^)	6 (5, 8)	8 (6, 10)	5 (5, 5)	5 (5, 5)	5 (5, 9)	31 (28, 35)
	KRUSKAL-WALLIS	*P*-value	**0.007**	**0.023**	0.148	** <0.001**	0.145	**0.013**
Sexually transmitted infections	NO (*n* = 361, 94.3%)	Med^a^ (Q1^b^,Q3^c^)	6 (5, 9)	8 (6, 11)	5 (5, 5)	5 (5, 6)	6 (5, 9)	32 (28, 38)
	YES (*n* = 15, 3.9%)	Med^a^ (Q1^b^,Q3^c^)	8 (6, 9)	10 (5, 12)	5 (5, 7)	6 (5, 7)	8 (6, 9)	36 (31, 41)
	MANN-WHITNEY	*P*-value	0.113	0.495	0.375	**0.042**	0.054	0.05
Number of sexual partners in the last year	0 (*n* = 64, 16.7%)	Med^a^ (Q1^b^,Q3^c^)	6 (5, 7)	9 (7, 11)	5 (5, 5)	5 (5, 6)	7 (5, 9)	33 (30, 40)
	1 (*n* = 271, 70.8%)	Med^a^ (Q1^b^,Q3^c^)	6 (5, 8)	8 (6, 10)	5 (5, 5)	5 (5, 6)	5 (5, 8)	31 (28, 36)
	2 (*n* = 27, 7.0%)	Med^a^ (Q1^b^,Q3^c^)	7 (5, 9)	9 (7, 11)	5 (5, 7)	5 (5, 6)	6 (5, 9)	33 (29, 44)
	3+ (*n* = 14, 3.7%)	Med^a^ (Q1^b^,Q3^c^)	7 (5, 10)	9 (7, 15)	5 (5, 6)	5 (5, 7)	7 (5, 11)	32 (29, 48)
	KRUSKAL-WALLIS	*P*-value	0.526	0.197	**0.037**	0.206	**0.001**	0.088
Abortion	NO (*n* = 294, 76.8%)	Med^a^ (Q1^b^,Q3^c^)	6 (5, 9)	8 (6, 11)	5 (5, 5)	5 (5, 6)	6 (5, 9)	31 (28, 38)
	YES (*n* = 18, 4.7%)	Med^a^ (Q1^b^,Q3^c^)	7 (5, 9)	9 (7, 12)	5 (5, 5)	6 (5, 8)	8 (5, 10)	36 (30, 46)
	MANN-WHITNEY	*P*-value	0.531	0.534	0.854	**0.032**	**0.031**	0.077

a *Med–median;*

b *Q1–first quartile;*

c *Q3–third quartile*.

*Values in bold indicate statistically significant results (p < 0.05)*.

Nearly all ACE scores (partial and total) were significantly higher in subjects who (Table 5): (a) attempted suicide; (b) committed self-harm; (c) reported being aggressive with an intimate partner. All ACE scores except *emotional neglect* were significantly higher in individuals who reported suffering violence from an intimate partner. Other correlations were found: of the 20 individuals who committed self-harm, 35% (*n* = 7) reported being violent with their intimate partners, while 26% (*n* = 5) reportedly were victims of intimate partner violence; and, of the 25 individuals who reported intimate partner violence perpetration, 40% (*n* = 10) also reported having suffered from violence from an intimate partner.

**Table 5:** Median, interquartile range, and p-value between the different ACE factors, self-harm behaviors, and perpetuation of violence.

		**Emotional abuse**	**Emotional neglect**	**Sexual abuse**	**Physical abuse**	**Physical neglect**	**CTQ-SF**	
Attempted suicide	NO (*n* = 360, 90.5%)	Med^a^ (Q1^b^,Q3^c^)	6 (5, 8)	8 (6, 10)	5 (5, 5)	5 (5, 6)	6 (5, 8)	31 (28, 37)
	YES (*n* = 24, 6.0%)	Med^a^ (Q1^b^,Q3^c^)	9 (7, 14)	11 (8, 17)	5 (5, 8)	6 (5, 8)	8 (5, 13)	43 (35, 56)
	MANN-WHITNEY	*P*-value	** <0.001**	**0.001**	** <0.001**	** <0.001**	** <0.001**	** <0.001**
Self-harm	NO (*n* = 308, 77.4%)	Med^a^ (Q1^b^,Q3^c^)	6 (5, 8)	8 (6, 11)	5 (5, 5)	5 (5, 6)	6 (5, 9)	31 (28, 37)
	YES (*n* = 20, 5.0%)	Med^a^ (Q1^b^,Q3^c^)	8 (7, 13)	11 (7, 17)	5 (5, 9)	7 (5, 8)	9 (5, 12)	41 (35, 56)
	MANN-WHITNEY	*P*-value	**0.001**	**0.029**	** <0.001**	** <0.001**	**0.006**	** <0.001**
Aggression against an intimate partner	NO (*n* = 295, 74.1%)	Med^a^ (Q1^b^,Q3^c^)	6 (5, 8)	8 (6, 11)	5 (5, 5)	5 (5, 6)	5 (5, 9)	31 (28, 36)
	YES (*n* = 25, 6.3%)	Med^a^ (Q1^b^,Q3^c^)	8 (7, 11)	10 (7, 17)	5 (5, 9)	7 (5, 8)	7 (6, 10)	41 (35, 54)
	MANN-WHITNEY	*P*-value	** <0.001**	**0.004**	**0.001**	** <0.001**	**0.002**	** <0.001**
Aggression by an intimate partner	NO (*n* = 306, 76.9%)	Med^a^ (Q1^b^,Q3^c^)	6 (5, 8)	8 (6, 11)	5 (5, 5)	5 (5, 6)	5 (5, 8)	31 (28, 37)
	YES (*n* = 15, 3.8%)	Med^a^ (Q1^b^,Q3^c^)	9 (5, 18)	10 (7, 15)	6 (5, 11)	6 (5, 8)	9 (7, 12)	47 (35, 61)
	MANN-WHITNEY	*P*-value	**0.007**	0.082	** <0.001**	**0.008**	** <0.001**	** <0.001**

a *Med–median;*

b *Q1–first quartile;*

c *Q3–third quartile*.

*Values in bold indicate statistically significant results (p < 0.05)*.

### Physical and Psychological Health and Correlations With ACE Factors

Table 6 illustrates the higher ACEs values for the considered pathologies in individuals who had the following pathologies: (a) individuals with hypercholesterolemia, HBP, or who had a stroke presented with higher partial *physical neglect* and total scores; (b) individuals with diabetes had significantly higher partial (emotional and physical neglect and physical abuse) and total ACE score; (c) individuals with rheumatoid arthritis exhibited higher partial (*emotional* and *physical neglect* and *sexual abuse*) and total ACE score; (d) individuals with an anxiety disorder had higher partial and total ACE score; (e) individuals with depression had higher partial (all except *emotional* and *sexual abuse*) and total ACE score.

**Table 6:** Absolute and relative frequency, median, and interquartile range of the scores of the different ACEs factors for each clinical condition and their respective *p*-values (Mann-Whitney test).

	**Emotional abuse Med^a^ (Q1^b^,Q3^c^)**	**Emotional neglect Med^a^ (Q1^b^,Q3^c^)**	**Sexual abuse Med^a^ (Q1^b^,Q3^c^)**	**Physical abuse Med^a^ (Q1^b^,Q3^c^)**	**Physical neglect Med^a^ (Q1^b^,Q3^c^)**	**CTQ-SF Med^a^ (Q1^b^,Q3^c^)**
**Cholesterol**, ***n*** **=** **109 (27.4%)**						
Absent	6 (5, 9)	8 (6, 10)	5 (5, 5)	5 (5, 6)	6 (5, 8)	31 (28,36)
Present	7 (5, 9)	9 (6, 12)	5 (5, 5)	5 (5, 6)	7 (5, 9)	34 (29,40)
*p*-value	0.718	0.157	0.45	0.416	**0.008**	**0.028**
**High blood pressure**, ***n*** **=** **93 (23.4%)**						
Absent	6 (5, 9)	8 (6, 11)	5 (5, 5)	5 (5, 6)	5 (5, 8)	32 (28,37)
Present	6 (5, 8)	9 (7, 11)	5 (5, 5)	5 (5, 6)	7 (5, 9)	35 (30,40)
*p*-value	0.748	0.13	0.678	0.1	** <0.001**	**0.044**
**Anxiety disorder**, ***n*** **=** **73 (18.3%)**						
Absent	6 (5, 8)	8 (6, 10)	5 (5, 5)	5 (5, 6)	6 (5, 8)	31 (28,26)
Present	7 (6,10)	9 (7, 14)	5 (5, 6)	5 (5, 7)	7 (5, 9)	35 (30,42)
*p*-value	**0.002**	**0.002**	**0.013**	**0.003**	**0.039**	** <0.001**
**Diabetes**, ***n*** **=** **61 (15.3%)**						
Absent	6 (5, 9)	8 (6, 10)	5 (5, 5)	5 (5, 6)	5 (5, 8)	31 (28,36)
Present	7 (5, 9)	9 (7, 12)	5 (5, 5)	5 (5, 7)	9 (6, 9)	35 (30,42)
*p*-value	0.928	**0.008**	0.741	**0.014**	** <0.001**	**0.001**
**Depression**, ***n*** **=** **59 (14.6%)**						
Absent	6 (5, 9)	8 (6, 10)	5 (5, 5)	5 (5, 6)	6 (5, 8)	31 (28,36)
Present	7 (5, 8)	9 (7, 13)	5 (5, 6)	5 (5, 7)	7 (5, 11)	36 (31,43)
*p*-value	0.383	**0.003**	0.07	**0.046**	** <0.001**	** <0.001**
**Rheumatoid arthritis**, ***n*** **=** **23 (5.8%)**						
Absent	6 (5, 9)	8 (6, 11)	5 (5, 5)	5 (5, 6)	6 (5, 8)	32 (28,38)
Present	8 (5, 9)	9 (8, 14)	5 (5, 6)	6 (5, 6)	9 (7, 11)	35 (33,46)
*p*-value	0.337	**0.04**	**0.009**	0.09	** <0.001**	**0.017**
**Stroke**, ***n*** **=** **20 (5.0%)**						
Absent	6 (5, 9)	8 (6, 11)	5 (5, 5)	5 (5, 6)	6 (5, 8)	32 (28,27)
Present	7 (6, 7)	9 (7, 13)	5 (5, 6)	5 (5, 6)	9 (6, 13)	35 (31,44)
*P*-value	0.481	0.138	0.301	0.067	** <0.001**	**0.029**
**Neoplasia**, ***n*** **=** **17 (4.3%)**						
Absent	6 (5, 8)	8 (6, 11)	5 (5, 5)	5 (5, 6)	6 (5, 8)	32 (28,38)
Present	6 (5, 9)	7 (6, 11)	5 (5, 5)	5 (5, 6)	9 (7, 11)	35 (29,40)
*p*-value	0.94	0.695	0.134	0.885	**0.01**	0.382

a *Med–Median;*

b *Q1–first quartile;*

c *Q3–third quartile*.

*Values in bold indicate statistically significant results (p < 0.05)*.

Positive moderated correlations were also found: (a) between depression/anxiety disorder and smoking dependence and (b) between depression/anxiety disorder and suicide attempts and/or self-harm behaviors.

## Discussion

In this study, we evaluated the impact of ACE/MT on adult individuals' health risk behaviors and their overall health. This is an original study, and no publications related to this impact on adult individuals or populations in the Porto region have been identified.

### Correlations Between ACE Factors and Respondent Characteristics

Regarding the respondents' age, we found a significant positive association with the *physical neglect* factor (Table 1). The explanation may lie in the fact that older individuals are more likely to have experienced physical neglect because it was socially acceptable and common when these respondents were children, given that the social condemnation and criminalization of such practices occurred only recently (Magalhães, 2020). We also found a significant but negative association between age and the *emotional abuse* factor (Table 1), which may reflect a greater awareness among younger individuals of what constitutes abuse; additionally, for temporal reasons, their self-reports may have greater reliability (Colman et al., 2016).

Regarding gender, we found that emotional and sexual abuse were more frequently reported by female respondents (Table 1), which is consistent with the literature that reports a higher frequency of females exposed to these types of abuse (Magalhães et al., 2010; Pinto et al., 2014).

### Health Risk Behaviors and Correlations With ACE

Adopting risk behaviors such as substance abuse as coping mechanisms by individuals with ACEs is widely described in the literature. A study by Anda et al. (2006) analyzed the relationship between the number of ACEs and 18 outcomes (including somatic disorders; consumption of tobacco, alcohol, and drugs; early onset of sexual activity; and multiple sexual partners). The relationship between the number of ACEs score and each of the outcomes under study, as well as with the total number of these outcomes, revealed a dose-response relationship, i.e., the higher the number of ACEs, the greater rate of the observed comorbidity. Similarly, the ACE Study (Felitti et al., 2019) concluded that individuals exposed to four or more ACEs' have an increased risk of alcoholism, drug abuse, depression, suicide attempts, tobacco use, multiple sexual partners, STI, physical inactivity, and severe obesity. The authors confirm the existence of a relationship between exposure to ACEs and the presence of several risk factors associated with the main causes of death in adults, i.e., early death. Additionally, a systematic review and meta-analysis by Hughes et al. (2017) defend that exposure to multiple ACEs is the greatest risk factor for various clinical conditions and risk behaviors, including a strong relationship between ACEs and alcohol abuse, substance use, risky sexual behavior, and mental illness.

The present study showed that individuals who have higher number ACEs have greater smoking dependence, similar to the findings of other studies (Dube et al., 2003; Anda et al., 2006; Mersky et al., 2013; Hughes et al., 2017). The explanation may be that nicotine has psychoactive properties (acting on the dopaminergic pathways) that promote increased pleasure sensation and decreased anxiety and lead to improved mood (Benowitz, 2010; Crouch et al., 2018).

No associations were found with other substance abuse, as would be expected (Cole et al., 2011; Mersky et al., 2013). This is probably due to the following: (1) it may be hypothesized that the sample was generally of medium-high-income population since it was collected in private health settings, and people with higher income are usually those with access to it (Nurses, 2011) and there are studies linking poverty or unemployment to alcohol use (Khan et al., 2002; Henkel, 2011) and low-income to lower illicit drug use (Sunder et al., 2007; Kuo et al., 2011), although other authors find access to drugs positively correlated to higher income (Long et al., 2014); (2) employment, a source of economic empowerment, is positively correlated to access to health services, especially private (Liu et al., 2013). (3) Substance abuse has been found to be more prevalent in lower social strata (Cole et al., 2011; Mersky et al., 2013). It is possible to hypothesize that accounting for family disfunction measures might allow to refine this correlation study.

Regarding the risky sexual behaviors that were analyzed, we found that some individuals who reported these behaviors had higher partial and total number of ACE. Additionally, a cohort found that each type of ACE was associated with an increased risk of early onset of sexual activity, multiple sexual partners, and HIV (Hillis et al., 2001). Regarding abortion in adolescence, another study evaluated its relationship with ACEs and concluded that, in addition to other factors, a history of ACEs (namely, physical and sexual abuse) is associated with repeated abortions (Fisher et al., 2005). Associations with sexual abuse were not found, which may be related to the low values (less respondents scoring in this type of abuse) and variability of responses found in the sexual abuse score. These values may occur due to the sample's social characteristics, as mentioned (Hillis et al., 2000), or due to stigmas in reporting sexual abuse.

Individuals who reported self-harm behavior had higher partial (except for sexual abuse) and total ACE scores. This relationship between self-harm behaviors and exposure to ACEs is consistent with the results of other authors who show that individuals exposed to ACEs have a higher risk of suicide attempts (Dube et al., 2003, 2005; Hughes et al., 2017). A cohort study by Dube et al. (2001) confirms that individuals exposed to ACE have an increased risk of suicide throughout their lives. This relationship is partially mediated by alcoholism, depressed mood, and substance abuse. These results suggest that ACEs are related to certain mental health outcomes (such as low self-esteem, emotional dysregulation, or inadequate attachment) that favor the development of depression and suicide attempts (Whiffen et al., 2000; Dube et al., 2001). However, it should be noted that the present study was conducted with a sample of adults who reported their childhood experiences, which, due to the elapsed time and the possible existence of other competing factors, hinders the establishment of a cause-effect relationship.

Results show that individuals who revealed that they had perpetrated or suffered violence in intimate relationships had higher ACE scores (except for sexual abuse in perpetrators) than those who did not commit or experience violence, pointing in the same direction as: (a) Heyman and Slep (2002), who found higher probability of experiencing and perpetrating partner abuse in those who had been exposed to adverse childhood experiences related to violence; (b) Anda et al. (2006) who found greater difficulty in anger control, and risk of perpetrating IPV, increased 4.0-,and 5.5-fold, respectively, for individuals with ≥4 ACEs and (3) Hughes et al. (2017), who found higher odds of both violence victimization and perpetration in individuals who had different ACEs.

There are significant associations between all factors except emotional neglect in victims. These results are in line with the systematic review and meta-analysis by Hughes et al. (2017), who argue that exposure to multiple ACEs is a risk factor for violence. It also considers that the outcomes most strongly associated with ACE represent an increased risk of these ACEs being transmitted to the next generation via generational transmission of violence, as it is referred to in the literature (Dodge et al., 1990; Pears and Capaldi, 2001; Heyman and Slep, 2002; Duke et al., 2010; Roberts et al., 2011).

Given these results, we can say that ACEs, as a source of stress, greatly impact the adoption of risk behaviors with harmful long-term consequences for the health of individuals who have experienced them.

### Physical and Psychological Health and Correlations With ACE

The most prevalent pathologies in the study sample were metabolic diseases (hypercholesterolemia and diabetes), cardiocerebrovascular diseases (HBP and stroke) and inflammatory diseases (rheumatoid arthritis) (Table 6).

The factor *physical neglect* had a statistically significant association with all of the diseases studied (Table 6), possibly because this factor is related to older age, as mentioned above (Table 1). Conversely, the negative correlation found between the *emotional abuse* factor and age (Table 6) may mask possible associations between this factor and certain health problems, as it is known that, as a rule, morbidity is higher at older ages. We did not find other associations, particularly with respiratory diseases and neoplasia; based on the literature, this may be expected due to the size of the sample (Dube et al., 2003; Anda et al., 2008; Holman et al., 2016; Felitti et al., 2019).

We found a hypercholesterolemia prevalence of 27.4%. When compared to a Portuguese population-based study that obtained a high low-density lipoprotein (LDL) cholesterol in of 63.3% (INSA, 2016), it may be hypothesized, since the latter considered not only self-reports (prevalence of 11%) but also biochemical analysis (prevalence of 52.3%). The laboratory confirmation detected hypercholesterolemia in individuals who did not know that they had it or did not report it.

The current study met a prevalence of 23.4% of HBP. Comparing to values previously found for the general Portuguese population aged 25 to 74 years (36%) this is much lower. Similarly to hypercholesterolemia, this discrepancy may be due to the fact that we based our research on self-reports alone, without a medical check. According to Stein et al. (2010), who analyzed the relationship between ACEs and early adult HBP, anxiety disorder and early-onset depression may be mediators of this relationship; moreover, they demonstrated that being exposed to one adverse event does not increase the risk of HBP in adulthood, while exposure to two or more ACEs increases this risk, and the reiteration and duration of events (dose-response effect) are relevant to the development of HBP.

In diabetes, a rate of 15.3% was found, which was higher than that found in the general Portuguese population (9.8%) (INSA, 2016). This rate agrees with the literature that predicts a higher risk of diabetes in individuals with ACEs (Huang et al., 2015; Huffhines et al., 2016).

The same trend was observed for rheumatoid arthritis, the 5.8% prevalence of which in the present study was also higher than that found in the Portuguese population (0.7%) (ReumaCensus, 2015). This finding is in line with the results from other studies, especially concerning women (Rubinstein et al., 2020). There are, however, studies that do not identify this relationship (Carette et al., 2000).

Anxiety disorder had a prevalence of 18.3%, slightly higher than a recent estimation made for the general Portuguese population (16.5%) (CNS, 2019). However, the possibility that current mental health problems influence how adversity experienced in childhood is manifested cannot be excluded (Colman et al., 2016). In this particular case, assessing the possible mediating role of household dysfunction might allow a more thorough approach. For depression, the prevalence was 15.3%, higher than the Portuguese prevalence rate (10%) (CNS, 2019), also suggesting higher rates in individuals reporting ACEs as in other studies (Poole et al., 2017; Von Cheong et al., 2017).

Suicidal attempts are known to be higher among those who report ACEs (Thompson et al., 2019), especially physical abuse (Fuller-Thomson et al., 2016; Thompson et al., 2019) which is confirmed by the present study.

There are findings supporting smoking-related DNA methylation changes due to toxic stress in early years suggesting greater nicotine consumption and dependence in individuals reporting ACEs (Sugden et al., 2019). In the present study, tobacco consumption was significantly related with reports of physical abuse while tobacco dependence is significantly associated with the global scale of trauma, with a highly significant association with neglect (emotional and physical) and emotional abuse.

Other findings of the current study are to be noted, although not depicted in the tables: (a) depression and anxiety disorder are have positive correlation with smoking dependence, which is aligned with other results (McKenzie et al., 2010). This dependence risk was found to be higher also in individuals who reported suicide attempts or self-harm behaviors, suggesting that it may be important to assess nicotine use in cases of suicide or self-harm related events (Yaworski et al., 2011).

The fact that no relationship was found between ACEs and other negative *outcomes* identified in the literature (Hillis et al., 2000; Dong et al., 2004; Mersky et al., 2013; Chiang et al., 2015; Holman et al., 2016; Felitti et al., 2019) may be related to some of the study's limitations discussed below.

### Study Limitations and Future Perspectives

We identified the following limitations in this study: (a) the limited number of cases for a population-based study; (b) the analysis of only five ACE factors, although the literature identifies additional factors of interest such as household ACEs (Felitti et al., 2019), considering that the focus of our study is MT; and, for that reason, experiences related to the death of a close relative, parental divorce, abuse of any family member and domestic violence were not analyzed, which might allow a more refined statistical analysis of findings; (c) specific ACEs such as household ACEs experienced by each participant were not evaluated, which might help understand the enduring effects of ACEs (Chapman et al., 2004; Anda et al., 2006; Hughes et al., 2017; Felitti et al., 2019), also limiting the extent of our interpretations; (d) possible bias related to some missing data (lack of answers), difficulty remembering or identifying certain ACEs (especially when they occurred at very early ages) and subjectivity/individual differences in the valuation of these experiences and the eventual incorrect classification of ACEs. A large number of these biases could be overcome with the use of an other-administered questionnaire, although this could create new biases, particularly those related to feelings of shame and difficulties with disclosure to others (Wojcik et al., 2019); (e) the fact that the sample was limited to the Porto region, and to a population that could afford private healthcare, which does not allow the generalization of the results to the entire country.

However, this study is the first consideration of this subject in the Porto region, and it should be considered a preliminary analysis.

Future studies should consider the limitations mentioned above, including increasing the sample size, analyzing and quantifying all of the identified ACEs, validating the I-CAST and its application to the Portuguese population, and possibly extending the studied geographic region.

## Conclusion

The results show significant associations between some (or all) ACE factors related to MT and specific adulthood characteristics. In particular, associations were found with health risk behaviors, including: smoking, early onset of sexual activity, STI, having multiple sexual partners in the last year, abortion, suicide attempts, self-harming behavior, victimization, and perpetration abuse in intimate relationships. Moreover, the ACE factors are related to pathologies, namely, hypercholesterolemia, stroke, HBP, diabetes, rheumatoid arthritis, neoplasia, depression, and anxiety disorder.

*Physical neglect* appears in this study as the ACE type that is most commonly found in individuals with the most prevalent health problems in the Portuguese population. All ACEs related to MT in this study are strongly related to violent experiences in adulthood, particularly violence in intimate relationships and self-directed violence.

Therefore, we identified the need to continue these studies in the various regions in Portugal to understand this phenomenon more broadly, given that it is essential for guiding prevention actions at various levels.

## Data Availability Statement

The original contributions presented in the study are included in the article. Further inquiries can be directed to the corresponding author.

## Ethics Statement

The studies involving human participants were reviewed and approved by Ethics Committee of the Hospital Centre of São João, Porto Portugal. The patients/participants provided their written informed consent to participate in this study.

## Author Contributions

TM and MN: conceptualization. MN and TH: methodology. MN, MV-A, and TH: formal analysis and investigation. MN, TM, and MV-A: writing—original draft preparation. TM, MV-A, and TH: writing—review and editing. All authors have read and agreed to the published version of the manuscript.

## Conflict of Interest

The authors declare that the research was conducted in the absence of any commercial or financial relationships that could be construed as a potential conflict of interest.
